# Physical activity substitution: An overlooked constraint on energy expenditure during exercise and physical activity interventions

**DOI:** 10.1111/dom.70079

**Published:** 2025-09-03

**Authors:** Dylan Thompson, Melina Del Angel, Matthew A. Nunes, Javier T. Gonzalez, James A. Betts, Oliver J. Peacock

**Affiliations:** ^1^ Centre for Nutrition, Exercise and Metabolism (CNEM), Department for Health University of Bath Bath UK; ^2^ Department of Mathematical Sciences University of Bath Bath UK

**Keywords:** compensation, constrained, exercise intervention, weight management

## Abstract

**Aims:**

When exercise is prescribed, the new exercise could ‘substitute’ for pre‐existing physical activity such that the net effect on energy expenditure is less than predicted. However, the impact of substitution has not been examined to date because of methodological spatiotemporal challenges. To overcome these challenges, we use mathematical modelling to examine the impact of substitution during prescribed exercise interventions.

**Materials and Methods:**

We modelled the impact of substitution during two prescribed exercise interventions (LOW and HIGH) on energy expenditure in 242 patients aged 63 ± 6 years recruited in the south‐west of England. The increase in net daily energy expenditure after subtraction of pre‐existing physical activity at the time of the new modelled prescribed interventional exercise was derived to account for the potential effect of substitution.

**Results:**

After accounting for substitution, the increase in daily energy expenditure was 38% ± 32% and 73% ± 12% of the potential increase under additive models for LOW and HIGH prescribed exercise scenarios, respectively. Furthermore, substitution introduced considerable heterogeneity in the predicted change in daily energy expenditure. This heterogeneity was most evident in the LOW prescribed exercise scenario, where the predicted change ranged from +91% to −93% of the anticipated increase under an additive model.

**Conclusions:**

Physical activity substitution has the potential to erode the increase in daily energy expenditure during prescribed exercise interventions, and to introduce marked heterogeneity in the treatment response (change in energy expenditure). Successful mitigation of physical activity substitution could potentially increase the effectiveness of physical activity interventions.

## 1 INTRODUCTION

Physical activity is often prescribed as a cornerstone in the treatment and management of chronic conditions such as cardiovascular disease and type 2 diabetes.^1^ However, when new physical activity is introduced, the new physical activity (e.g., prescribed exercise) will be superimposed against the background of pre‐existing daily life. Thus, pre‐existing/baseline energy expenditure at the time of the new physical activity will not always be equivalent to rest. Instead, the new physical activity may sometimes ‘substitute’ for pre‐existing physical activity such that the net change in energy expenditure is not what would be predicted based on the addition of new prescribed (interventional) gross energy expenditure. Surprisingly, there has been little research on the impact of physical activity substitution on energy expenditure during interventions. Part of the reason for this omission may be because it is challenging to empirically determine and quantify the impact of substitution. Such an experiment would rely on the ability to simultaneously quantify what new physical activity energy expenditure has been introduced whilst also knowing what physical activity would have otherwise naturally occurred at precisely the same time under free‐living conditions.

Randomised Controlled Trials (RCTs) using robust techniques such as doubly labelled water often report that the increase in measured total energy expenditure from exercise and physical activity interventions falls short of what would be predicted based on the addition of the prescribed exercise, even when the new physical activity behaviour has been directly supervised.^2^, ^3^, ^4^ This is often interpreted as evidence for the existence of compensatory behavioural and/or biological metabolic responses that serve to constrain energy expenditure (i.e., where the introduction of new physical activity causes a decrease in physical activity at other times of the day or week).^4^, ^5^, ^6^, ^7^, ^8^ By extension, it has been suggested that compensatory behavioural and/or metabolic responses may partially explain why weight loss from physical activity/exercise interventions is often less than predicted from additive energy expenditure models.^2^, ^5^ However, such conclusions overlook the potential impact from physical activity substitution. Some of the failure of additive energy expenditure models to ‘add up’ could be because the sum is incomplete (i.e., missing a subtraction component for the displaced physical activity energy expenditure), and not necessarily due to a compensatory behavioural or biological response.

In addition to affecting energy expenditure, physical activity substitution may also contribute to heterogeneity in weight loss following exercise interventions. Even very carefully supervised exercise interventions report heterogeneity in weight loss, which is often depicted with waterfall plots showing individual variability in response to the same prescribed exercise energy expenditure.^9^, ^10^, ^11^ It is often speculated that such heterogeneity is caused by variation in compensatory behavioural changes to habitual non‐exercise physical activity and/or compensatory increases in energy intake.^12^, ^13^ However, an alternative and/or complementary possibility is that some of this heterogeneity is attributable to variability in the magnitude of physical activity substitution.

The lack of previous research into physical activity substitution is a major omission. Since it is not possible to directly assess substitution during interventions, the aim of the current study is to use mathematical modelling to explore the potential impact of physical activity substitution on the predicted net change in energy expenditure during exercise interventions.

## 2 MATERIALS AND METHODS

### 2.1 Design and approach

Technological advances in the availability of devices to measure free‐living physical activity make it possible to capture minute‐by‐minute records of daily physical activity over an extended period. Using such records, it is possible to model the theoretical impact of the addition of new prescribed exercise after accounting for the potential effects of substitution. In this report, we model the impact of two contrasting prescribed exercise scenarios (LOW and HIGH) in a group of patients recruited via primary care.

### 2.2 Participants and source data

This study uses data from the Multidimensional Individualised Physical Activity (MIPACT) randomised controlled trial.^14^, ^15^ MIPACT recruited patients aged 40 to 70 years at medium or high risk of cardiovascular disease and/or type II diabetes via primary care in the United Kingdom. Participant characteristics are shown in Table 1.

**TABLE 1:** Characteristics of study participants (*N* = 242).

Variable	
Age (years)	63 (6)
Male sex	157 (65)
Employment status	
In full time or part‐time employment	107 (44)
Retired	135 (56)
Area deprivation (IMD score)	7.7 (2.3)
Education levels	
Up to age 16 or less	72 (30)
Up to age 18	72 (30)
Undergraduate/higher degree	98 (40)
Current smoker	25 (10)
Body mass (kg)	83.7 (14.7)
Body mass index (kg/m^2^)	28.6 (4.5)
Waist circumference (cm)	98.9 (11.0)

*Note*: Data are expressed as mean (SD) for continuous data, or *N* (%) for categorical data.

Abbreviation: IMD, index of multiple deprivation.

In MIPACT, physical activity was assessed using the BodyMedia Sensewear® Core device (Pro 8.0, algorithm v5.2, BodyMedia Inc., Pittsburgh, PA). This multisensor wearable device is worn continuously on the upper arm. This device accurately measures minute‐by‐minute energy expenditure relative to criterion measurements,^16^, ^17^, ^18^ and thus provides sufficient resolution to undertake mathematical modelling. To model substitution over the full range of physical activity, we include baseline physical activity data for participants screened out of the MIPACT trial who had a high Physical Activity Level (PAL) greater than 2.0.^14^, ^15^ We exclude people who did not have a full 7‐day physical activity record (*N* = 10). This resulted in a total sample size of *N* = 242 for the current analysis.

Physical Activity Level (PAL) and wear time are shown in Table 2. PAL ranged from 1.3 to 2.7, which represents the typical range for PAL.^20^ Total average wear time was very high at almost 99%, and thus the physical activity record contains very little missing data (Table 2). The primary outcome in the MIPACT randomised controlled trial was the change in energy expenditure (PAL), but the intervention also included other secondary outcomes such as changes in fasting glucose and insulin.^14^, ^15^

**TABLE 2:** PAL and wear time for the MIPACT cohort.

Variable	Mean (SD)	Range
PAL	1.77 (0.26)	1.32–2.70
Wear time (%)	98.6 (1.4)	85.6–100.0

*Note*: Wear time over the 7‐day period of observation (%). Data are expressed as mean (SD) with ranges.

Abbreviation: PAL, Physical Activity Level (Total Energy Expenditure/Basal Metabolic Rate from Schofield equations^19^).

### 2.3 Modelling

We modelled the addition of new physical activity according to two contrasting theoretical scenarios: LOW and HIGH. The LOW scenario represents the addition of 150 min per week of new physical activity at an intensity of 3 metabolic equivalents (METs), divided into 3 bouts of 50 min. One MET is considered equivalent to basal metabolic rate.^21^ The HIGH dose scenario represents the addition of 300 min per week of new physical activity at an intensity of 6 METs, divided into 5 bouts of 60 min. Thus, LOW and HIGH represent variable volumes of moderate and vigorous intensity physical activity defined using MET intensity thresholds.^22^, ^23^

We utilised Python (version 3.11.3) to process 7‐day raw energy expenditure records. Energy expenditure data, at minute resolution, was pre‐processed to impute missing values from the small amount of non‐wear time using the lowest recorded energy expenditure as a measure of basal metabolic rate. We used the lowest recorded energy expenditure from the device (rather than Schofield equation estimates of basal metabolic rate) to maintain coherence between the modelled data and the original source data, and to avoid negative values. The lowest metabolic rate was 0.07 ± 0.07 kcal/min lower than Schofield estimated basal metabolic rate.

The theoretical impact of new physical activity was added to pre‐existing daily energy expenditure to derive the estimated net increase in daily energy expenditure under a simple additive model (i.e., where all the energy expended during new physical activity is added to pre‐existing energy expenditure). This represents the Theoretical Additive condition (no substitution). For example, for someone with a basal metabolic rate of 1 kcal/min, 150 min a week at 3 METs (LOW) is equivalent to a 300‐kcal net increase in weekly energy expenditure (after adjustment for basal metabolic rate over this 150‐min period). This would theoretically add (increase) daily energy expenditure by 43 kcal/d for this individual. The HIGH scenario would theoretically increase net daily energy expenditure by 214 kcal/d for this individual.

**FIGURE 1:**
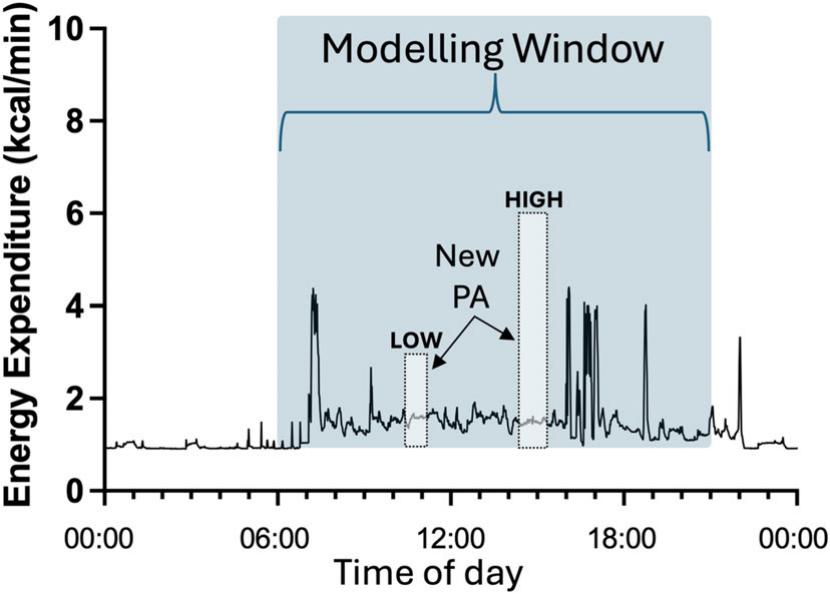
An example 24 h record for energy expenditure data along with the time window that was used for modelling (600–2100 h). New physical activity (PA) energy expenditure under LOW or HIGH modelled scenarios was added randomly during the highlighted modelling window (see text for details).

To model the impact of substitution, in both scenarios, LOW or HIGH bouts were placed at random to start after 6:00 AM and finish by 9:00 PM on different days, replacing pre‐existing energy expenditure data (Figure 1). Random placement was performed in *R* (version 4.3.0),^24^ using the built‐in function *sample*() that generates random samples. This function was used to uniformly select eligible start times within the defined time window. Sampling was conducted without replacement to ensure that there was no overlap between bouts. The estimated increase in net daily energy expenditure after subtraction of pre‐existing physical activity at the time of the new modelled prescribed interventional exercise was derived to account for the potential effect of substitution. This represents the Modelled Additive condition (with substitution).

## 3 RESULTS

### 3.1 Theoretical additional energy expenditure

**FIGURE 2:**
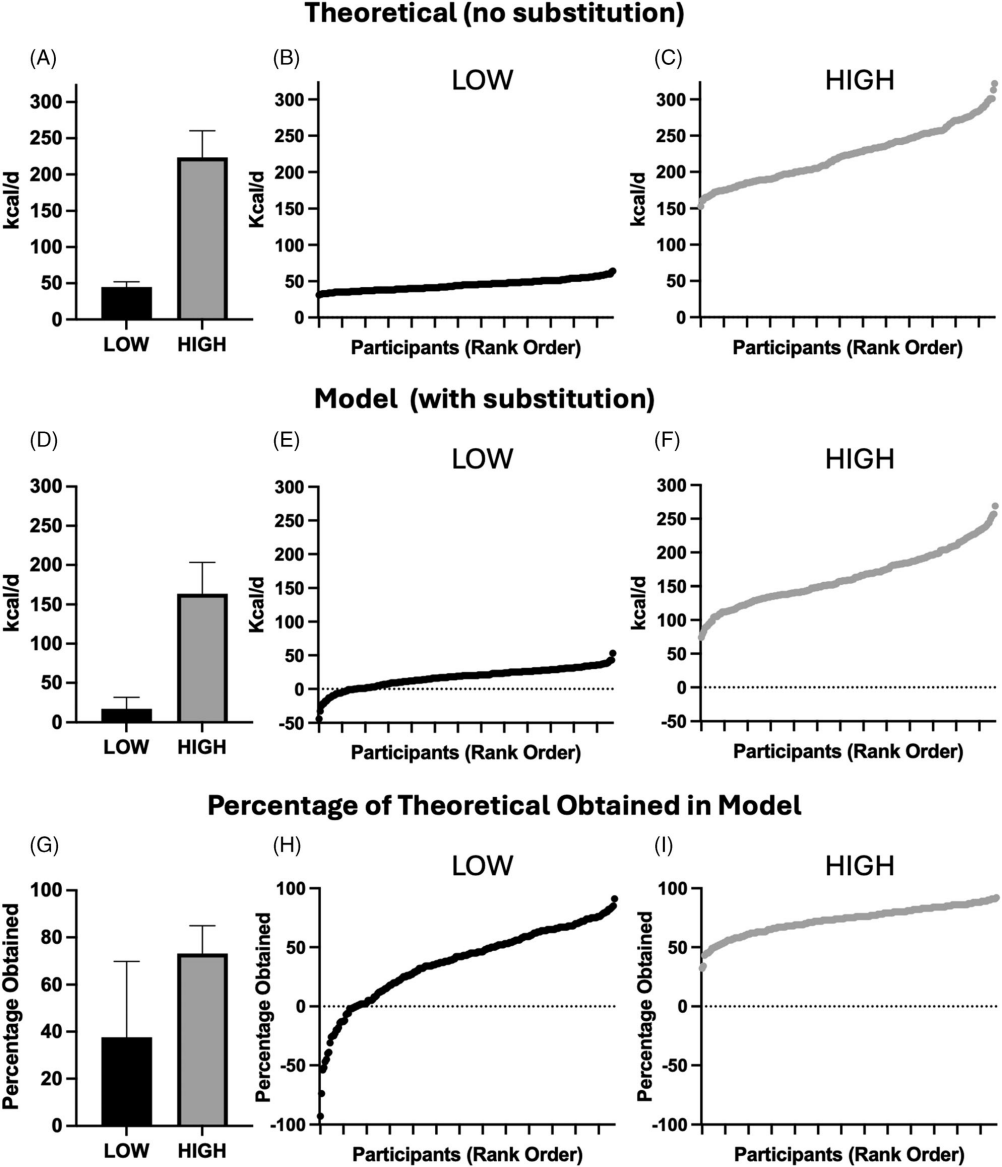
The predicted change in daily energy expenditure under LOW and HIGH dose prescribed exercise scenarios. *Theoretical Additive* represents the simple addition of new prescribed exercise energy expenditure to pre‐existing energy expenditure at group (A) and individual levels (B, C). *Modelled Additive* shows group (D) and individual data (E, F) after adjustment for the impact of substitution. The *Percentage of Theoretical Additive Obtained* in Modelled Additive is shown at group (G) and individual levels (H, I). Data in Panels A, D, and G represent the mean (SD) for *N* = 242. The data in Panels B, C, E, F, H, and I are individual data in rank order for LOW and HIGH scenarios, respectively.

In LOW and HIGH scenarios, the mean theoretical increase in daily energy expenditure from new prescribed exercise under a simple additive model (i.e., without accounting for the impact of substitution) was 45 ± 7 and 224 ± 37 kcal/d, respectively (Figure 2A). The data for LOW and HIGH Theoretical Additive scenarios at the individual level is shown by rank order in Figure 2B,C, respectively.

### 3.2 Modelled additional energy expenditure after accounting for substitution

After accounting for the effect of substitution, the mean increase in energy expenditure in the LOW Modelled Additive scenario was 17 ± 15, and 164 ± 40 kcal/d in the HIGH scenario (Figure 2A). This represents 38% ± 32% and 73% ± 12% of the potential Theoretical Additive model for LOW and HIGH scenarios, respectively (Figure 2G).

There was considerable heterogeneity for both LOW and HIGH Modelled Additive scenarios at the individual level (Figure 2E,F,H,I). In every individual case, across both LOW and HIGH scenarios, the Modelled Additive increase in energy expenditure was less than predicted under the Theoretical Additive model. The highest proportion of Theoretical Additive obtained in the LOW scenario was 91% (Figure 2H). Notably, in 12% of individuals (*N* = 29) in the LOW scenario, the impact of substitution *decreased* energy expenditure (Figure 2E,H). In the HIGH Modelled Additive scenario, the addition of new physical activity always increased energy expenditure (Figure 2F,I). In the HIGH scenario, the lowest individual increase as a proportion of Theoretical Additive was 32%, and the highest individual increase was 92% (Figure 2I).

The effect of Theoretical Additive and Modelled Additive scenarios on overall daily Physical Activity Energy Expenditure (PAEE) is shown in the Supporting Information (Figure S1).

### 3.3 Relationship between the degree of substitution and pre‐existing PAEE

**FIGURE 3:**
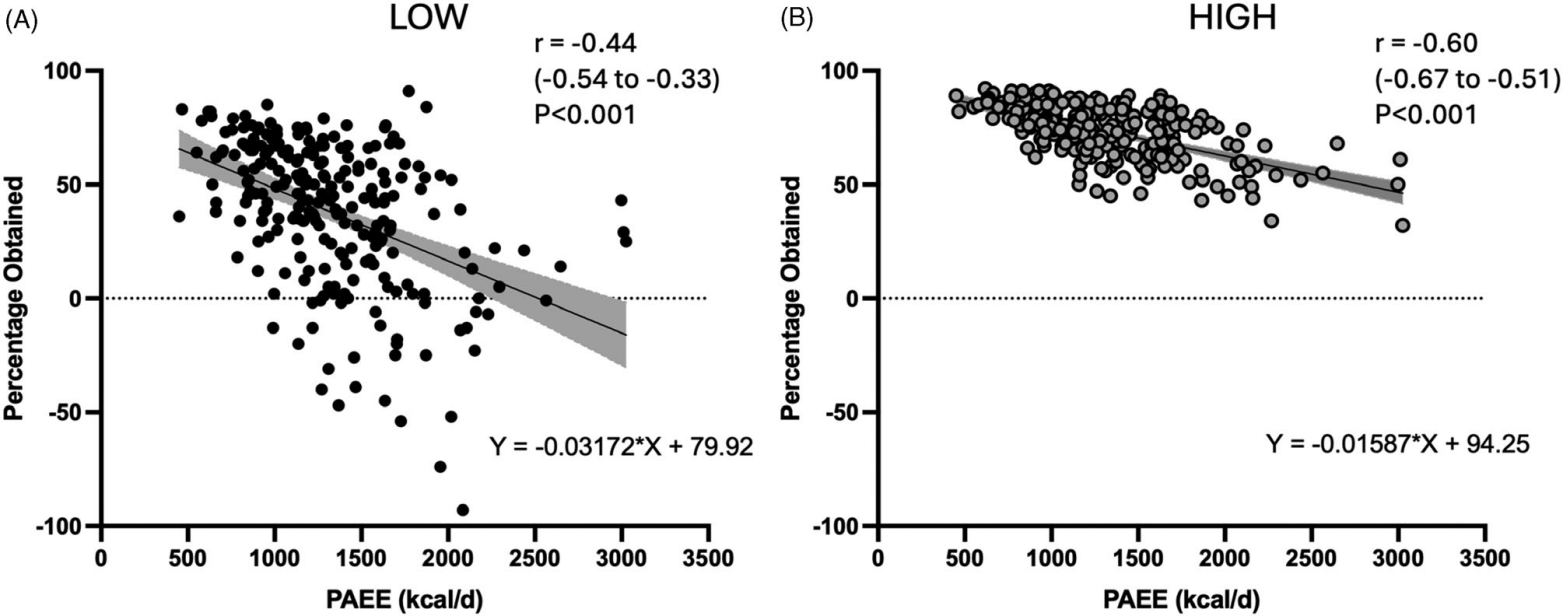
The relationship between the percentage of Theoretical Additive achieved in Modelled Additive and physical activity energy expenditure (PAEE) in LOW (A) and HIGH scenarios (B). Each point represents an individual (*N* = 242). The regression lines with 95% CIs are included. Pearson's correlation plus 95% CIs are also shown with the associated *p* value.

We explored the relationship between the change in energy expenditure under the Modelled Additive scenario (percentage of theoretical obtained) and habitual Physical Activity Energy Expenditure (PAEE) at the individual level (Figure 3). There was a negative relationship in both LOW and HIGH scenarios. This indicates that the effect of substitution is generally strongest when habitual PAEE is higher. Notably, the impact of substitution introduced markedly more variability in the LOW scenario (Figure 3A).

## 4 DISCUSSION

We demonstrate that physical activity substitution is a major consideration during prescribed exercise interventions. The addition of new prescribed exercise energy expenditure will rarely produce an equivalent increase in total energy expenditure because of the effects of physical activity substitution. The impact of substitution was particularly pronounced (and more variable) in the LOW prescribed exercise scenario.

The average increase in energy expenditure in the LOW scenario was less than half (38%) of what might be predicted and, for a small number of people, energy expenditure would be predicted to decrease. In the HIGH scenario, the impact of physical activity substitution was still considerable, but the net effect on overall energy expenditure was always positive (+73% on average, range 32%–92%). Thus, physical activity substitution could readily explain why the increase in daily energy expenditure is less than predicted during exercise interventions^2^, ^3^, ^4^, ^25^ and, as a corollary, why weight loss even during carefully controlled and supervised exercise interventions is generally less than expected.^25^ Substitution could also explain some of the apparent heterogeneity between people in response to physical activity interventions, including reports of weight gain in some people.^2^, ^5^, ^9^ Thus, physical activity substitution can have quantitatively meaningful effects on the potential change in energy expenditure during intervention studies and, by extension, on energy balance. Consequently, physical activity substitution is a major consideration during prescribed exercise interventions alongside other issues such as behavioural and biological compensation.

Physical activity substitution may also contribute to previous reports that prescribed exercise does not increase daily energy expenditure or physical activity, even when participation in exercise training has been directly supervised and observed.^2^, ^26^, ^27^, ^28^, ^29^ The lack of an increase in physical activity or PAEE in these studies may not necessarily be due to behavioural compensation in other non‐prescribed physical activity. Instead, it could be that the new exercise (e.g., a prescribed bout of walking) simply replaced non‐prescribed (existing) physical activity of a similar intensity with a resultant negligible effect on total energy expenditure. In the current analysis, the LOW prescribed scenario was similar to the minimum weekly physical activity recommendation (150 min at 3 METs). After accounting for substitution, this prescribed physical activity would only modestly increase daily energy expenditure by ~17 kcal/d in men and women aged 40–70 years recruited through primary care.

Physical activity substitution also has implications for heterogeneity in treatment response(s). Heterogeneous responses to the same carefully controlled exercise intervention, and even so‐called adverse responses,^30^ could be due to heterogeneity in the actual dose that was achieved once the (variable) effects of physical activity substitution have been considered. After accounting for the effect of substitution, the LOW scenario would change daily energy expenditure from a −33 kcal/d decrease to a +53 kcal/d increase. Thus, carefully standardised and delivered supervised exercise will not necessarily represent the same net dose across individuals because of variable degrees of physical activity substitution.

**FIGURE 4:**
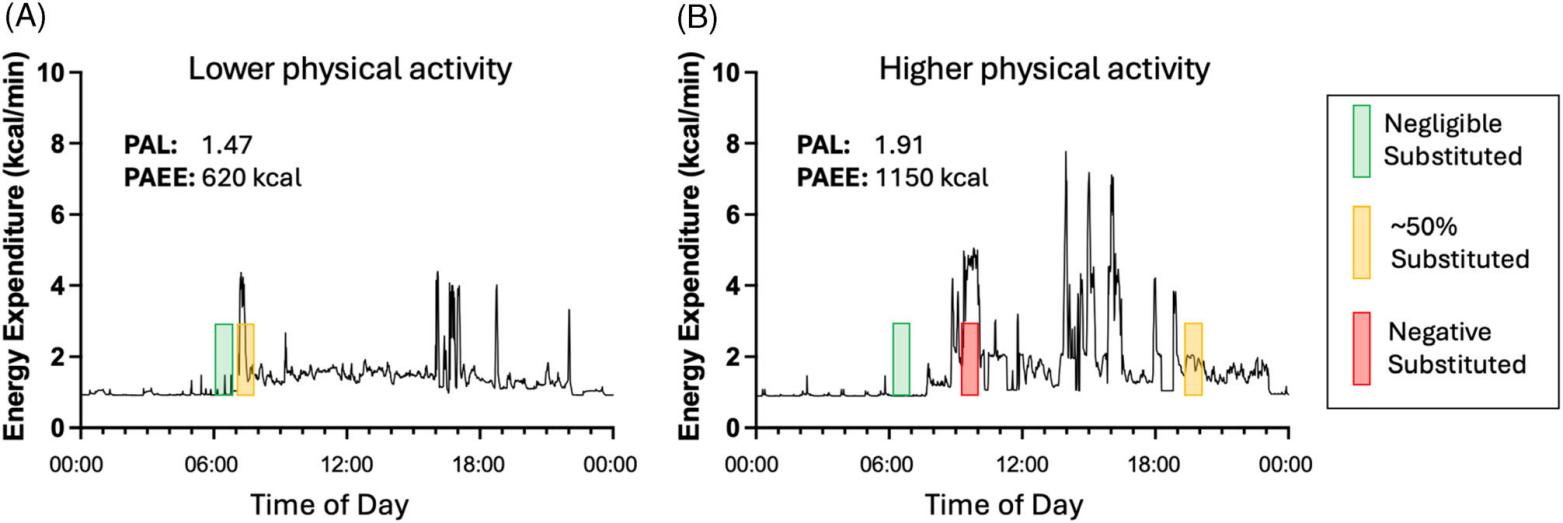
An illustration of the potential impact of physical activity substitution in a person with lower (A) and higher (B) habitual physical activity. A new bout of exercise equivalent to the LOW scenario (3 METs for 50 min) is depicted using the shaded rectangle at different times of day to illustrate the potentially heterogenous effects of substitution dependent on the timing of the new exercise/physical activity. The green shading indicates the placement of new exercise at a time that displaces little or no existing physical activity (negligible substitution) such that daily energy expenditure would increase by the full amount expended during the new exercise. The amber shading indicates the placement of new exercise that would increase energy expenditure by ~50% of that anticipated due to the effect of physical activity substitution. The red shading indicates the placement of new exercise at a time that displaces pre‐existing physical activity of a higher intensity than the new exercise such that daily energy expenditure would actually decrease.

The impact of substitution was greatest when habitual daily PAEE was higher. The potential effects are illustrated in Figure 4. As indicated, the new prescribed exercise could displace rest (green) or various amounts of pre‐existing physical activity (amber/red). Differences in the amount and distribution of pre‐existing habitual PAEE will influence the magnitude of substitution. In the HIGH scenario, the impact of substitution was reasonably consistent and never detrimentally affected daily energy expenditure. The relationship was more heterogeneous in the LOW scenario, which is probably because the new exercise is more likely to displace some equivalent (and sometimes higher) pre‐existing physical activity (Figure 4). Many activities of daily living are performed at an intensity similar to or higher than the LOW prescribed scenario (e.g., cleaning).^31^ Notably, people with low habitual physical activity will still be prone to some effect from substitution (Figure 4A). Indeed, even in settings that would be considered highly sedentary (a metabolic chamber), there will often be some physical activity that could be ‘substituted’.^32^, ^33^

This analysis demonstrates that the application of simple additive energy expenditure models to estimate the impact of new physical activity will be limited because they do not account for the pre‐existing energy expenditure that will be ‘lost’. Deriving an appropriate subtrahend to reflect the displaced energy expenditure will not be a trivial task. Depending on the study, it may be possible to use linear regression with modelling for the specific intervention that has been implemented similar to that used in the present study. In the HIGH scenario, based on our regression analysis, a person with a PAEE of 500 kcal/d would be anticipated to obtain 85% of the theoretical increase in daily energy expenditure from the intervention, whereas someone with a PAEE of 1500 kcal/d would obtain only 70%. Thus, modelling using participants' pre‐intervention PAEE may be one way to estimate the potential impact of substitution. It may even be possible to build in additional prescribed exercise to offset the physical activity that is anticipated to be lost. This approach would be challenging or impossible for the LOW scenario given the much more heterogeneous and unpredictable response. An alternative approach may be to estimate the impact of substitution retrospectively based on mathematical modelling of longitudinal time series energy expenditure data prior to the intervention. Such models may be able to estimate or predict what physical activity has been likely to have been ‘lost’ during the prescribed interventional exercise based on longitudinal historical patterns in daily energy expenditure.^34^ Whilst these approaches are unlikely to be perfect, they should be better than simply assuming that the new exercise or physical activity replaces complete rest.

It is important to note that this analysis reports the *potential* for substitution, and of course it cannot determine what would actually happen under real‐world conditions. This analysis assumes that all the displaced (substituted) physical activity is lost (i.e., that the substituted physical activity is completely discretionary). This is likely to be an overestimate because some of the displaced physical activity may be reallocated to another part of the day or week. For example, this might happen if some of the displaced activity was a necessary chore or task (e.g., walking a dog). However, many activities will be discretionary and not automatically reallocated—and some necessary activities may be allocated to other people (e.g., a family member). It is also possible that the loss of PAEE could be further compounded by the separate issue of behavioural activity compensation.^35^ Potentially, both substitution and compensation could be mitigated through self‐monitoring of physical activity using wearable devices. For example, we previously proposed that people could use wearable devices to identify regular periods of low PAEE which might represent an ideal time to insert new behaviours.^14^, ^15^ Furthermore, individuals could use wearable devices to understand the net effect on energy expenditure from the introduction of a new behaviour.^14^, ^15^ Thus, whilst substitution is a challenge for researchers, at an individual level, it could potentially be managed with the utilisation of appropriate wearable technologies. Further work can seek to understand if a substitution‐informed intervention linked to wearable devices helps overcome the potential detrimental impact of physical activity substitution (e.g., targeting habitually sedentary periods).

This study has many strengths. The source data represent a large sample of men and women recruited through primary care. The device and software that was used to assess energy expenditure have been extensively validated^16^, ^17^, ^18^, ^36^ and is classified by the US Food and Drug Administration (FDA) as a Class II medical device. Furthermore, the dataset was almost complete, with very little missing data (~1%). The range of physical activity in this sample spans the typical range for PAL (1.3–2.7), with average PAL a little higher than the estimated UK median PAL of 1.63.^37^ Our LOW modelled scenario is equivalent to the lower end of most international recommendations (150 min/week at 3 METs). This intensity is broadly equivalent to slow or leisurely walking.^31^ The HIGH scenario is equivalent to vigorous intensity physical activity using absolute MET thresholds and is similar to what would be considered a high dose in exercise RCTs.^2^, ^4^ Other studies should seek to explore the impact of substitution under different scenarios—including different populations, settings, and exercise interventions. For example, it could be useful to model the impact of high‐intensity time‐efficient interventions given the much smaller time window for pre‐existing physical activity to be substituted. It is possible that some interventions may still have a net positive effect on some health outcomes even if there is substitution (e.g., resistance exercise). Future research should look to examine the impact of substitution using a range of different physical activity interventions. It may also be useful to factor in some kind of quotient for estimated post‐exercise energy expenditure under different scenarios. The current study only examined direct substitution without considering any post‐exercise change in energy expenditure. We also set the modelling window as a fixed period (6:00 AM to 9:00 PM). The early start and late finish were to account for the possibility that people might try to incorporate new exercise at any time, including before or after work. Future studies could try to link substitution to qualitative data collected from individuals regarding their likely strategy for the incorporation of new exercise. For example, it may be possible to improve the modelled impact of substitution if study participants have a preconceived notion of when they would be likely to undertake new prescribed exercise. It may also be possible to improve modelling outcomes after accounting for documented individual‐level sleep–wake cycles, and/or with the addition of qualitative data from diaries to understand what specific pre‐existing activities have been substituted, and whether some activities have been or would be reallocated. One limitation to the analysis is that the algorithms used by the device are proprietary and the underlying assumptions are unknown. For example, the algorithms must account for Dietary Induced Thermogenesis (DIT) to estimate total daily energy expenditure, but it is not clear how DIT is incorporated. It is likely that DIT is incorporated into the PAEE component in our analysis. DIT is generally considered to be equivalent to 10% of total energy expenditure.^38^ This will not affect differences between Theoretical and Modelled Additive comparisons, but overall daily PAEE is likely to be a little high due to DIT being included in this component. Future studies might like to explore the issue of substitution using a range of different devices and algorithms.

In conclusion, physical activity substitution is a major consideration during prescribed exercise interventions. Substitution erodes the potential increase in daily energy expenditure and may even decrease energy expenditure in some situations. Thus, simple additive energy expenditure models should not be used to predict the impact of new exercise interventions on energy expenditure or energy balance. Given technological innovation, there are novel opportunities to use longitudinal data from wearable devices with appropriate predictive modelling to potentially mitigate the effects of substitution and thus increase the effectiveness of physical activity interventions.

## AUTHOR CONTRIBUTIONS

*Conceptualisation*: Dylan Thompson, Melina Del Angel, Matthew A. Nunes, James A. Betts, Javier T. Gonzalez and Oliver J. Peacock. *Data curation*: Dylan Thompson and Melina Del Angel. *Formal analysis*: Dylan Thompson and Melina Del Angel. *Funding acquisition*: Dylan Thompson. *Investigation*: Dylan Thompson, Melina Del Angel and Oliver J. Peacock. *Methodology*: Dylan Thompson, Melina Del Angel, Matthew A. Nunes and Oliver J. Peacock. *Project administration*: Dylan Thompson. *Resources*: Dylan Thompson. *Software*: Melina Del Angel. *Supervision*: Dylan Thompson and Matthew A. Nunes. *Validation*: Dylan Thompson, Melina Del Angel and Oliver J. Peacock. *Visualisation*: Dylan Thompson and Melina Del Angel. *Writing—original draft*: Dylan Thompson and Melina Del Angel. *Writing—review and editing*: All authors.

## CONFLICT OF INTEREST STATEMENT

James A. Betts is an investigator on research grants funded by BBSRC, MRC, NIHR, British Heart Foundation, Rare Disease Foundation, EU Hydration Institute, GlaxoSmithKline, Nestlé, Lucozade Ribena Suntory, ARLA Foods, Cosun Nutrition Center, American Academy of Sleep Medicine Foundation, Salus Optima (L3M Technologies Ltd), and the Restricted Growth Association; has completed paid consultancy for PepsiCo, Kellogg's, SVGC, and Salus Optima (L3M Technologies Ltd); is Company Director of Metabolic Solutions Ltd.; receives an annual honorarium as a member of the academic advisory board for the International Olympic Committee Diploma in Sports Nutrition; and receives an annual stipend as Editor‐in‐Chief of the *International Journal of Sport Nutrition & Exercise Metabolism*. Javier T. Gonzalez has received research funding from BBSRC, MRC, British Heart Foundation, Clasado Biosciences, Lucozade Ribena Suntory, ARLA Foods Ingredients, Cosun Nutrition Center, and the Fruit Juice Science Centre; is a (non‐exec) scientific advisory board member to ZOE; and has completed paid consultancy for 6d Sports Nutrition, The Dairy Council, PepsiCo, Violicom Medical, Tour Racing Ltd., and SVGC. For a full list of disclosures see https://gonzalezjt1.wordpress.com/2024/03/. The other authors have no potential conflicts of interest to declare.

## PEER REVIEW

The peer review history for this article is available at https://www.webofscience.com/api/gateway/wos/peer‐review/10.1111/dom.70079.

## Supporting information


**Figure S1.** The effect of additional physical activity on PAEE. Actual represents observed daily PAEE in 242 men and women. ‘Theoretical Additive, No substitution’ represents the simple addition of ‘new’ physical activity according to LOW (A) and HIGH (B) scenarios, whereas ‘Modelled Additive, with substitution’ represents PAEE after accounting for the modelled effect of substitution.

## Data Availability

The data reported in this paper are available through the University of Bath Data Archive (https://doi.org/10.15125/BATH-01591).
