# Supplementary material for: Physical activity substitution: An overlooked constraint on energy expenditure during exercise and physical activity interventions
Source: Diabetes Obes Metab. 2025 Sep 3;27(11):6682–90. doi: 10.1111/dom.70079 (PMC12515778; doi:10.1111/dom.70079)
Supplement: Supplementary file 1 — Figure S1. The effect of additional physical activity on PAEE. Actual represents observed daily PAEE in 242 men and women. ‘Theoretical Additive, No substitution’ represents the simple addition of ‘new’ physical activity according to LOW (A) and HIGH (B) scenarios, whereas ‘Modelled Additive, with substitution’ represents PAEE after accounting for the modelled effect of substitution. [file DOM-27-6682-s001.pdf]

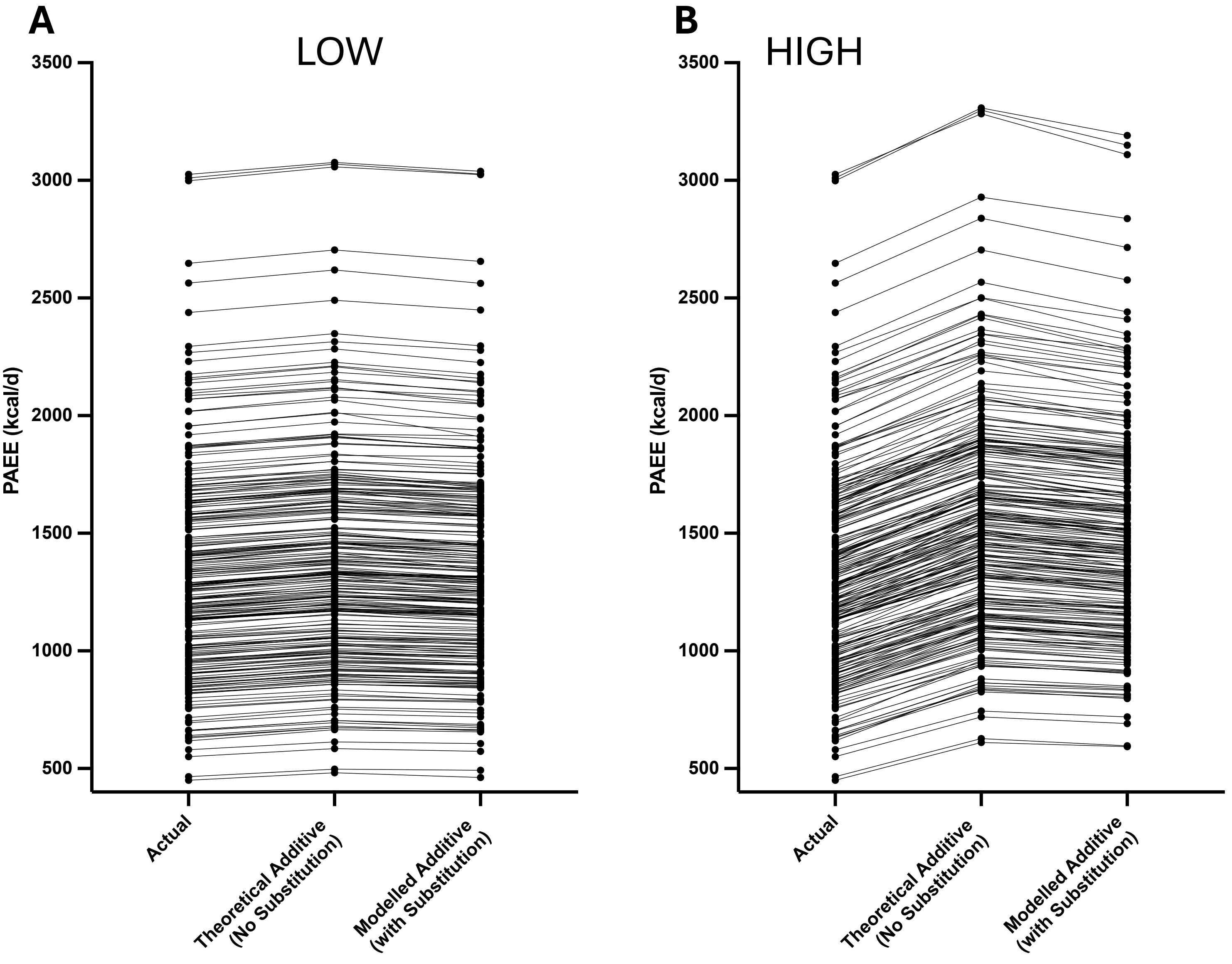


**Figure S1:** The effect of additional physical activity on PAEE. Actual represents observed daily PAEE in 252 men and women. 'Theoretical Additive, No substitution' represents the simple addition of 'new' physical activity according to LOW (A) and HIGH (B) scenarios, whereas 'Modelled Additive, with substitution' represents PAEE after accounting for the modelled effect of substitution.
